# Autophagy inhibition enhances photocytotoxicity of Photosan-II in human colorectal cancer cells

**DOI:** 10.18632/oncotarget.14117

**Published:** 2016-12-23

**Authors:** Li Xiong, Zhipeng Liu, Guoqing Ouyang, Liangwu Lin, He Huang, Hongxiang Kang, Wei Chen, Xiongying Miao, Yu Wen

**Affiliations:** ^1^ General Surgery Department of Second Xiangya Hospital, Central South University, Changsha, HN, China; ^2^ China State Key Laboratory for Powder Metallurgy, Central South University, Changsha, HN, China; ^3^ Department of Histology and Embryology, Xiangya School of Medicine, Central South University, Changsha, HN, China; ^4^ Institute of Radiation Medicine, Academy of Military Medical Sciences, Beijing, China; ^5^ Department of Physics, University of Texas at Arlington, Arlington, TX, USA

**Keywords:** apoptosis, autophagy, photodynamic therapy, colorectal cancer, Photosan-II

## Abstract

Photodynamic therapy (PDT) has emerged as an attractive therapeutic treatment for colorectal cancer because of its accessibility through endoscopy and its ability to selectively target tumors without destroying the anatomical integrity of the colon. We therefore investigated the therapeutic relevance of the interplay between autophagy and apoptosis in Photosan-II (PS-II)-mediated photodynamic therapy (PS-PDT) in *in vitro* and *in vivo* models for human colorectal cancer. We observed that PS-PDT-induced dose-dependently triggered apoptosis and autophagy in both SW620 and HCT116 cells. PS-PDT-treated SW620 cells exhibited nuclear condensation and increased levels of cleaved caspase-3, PARP and Bax, which is reminiscent of apoptosis. PS-PDT also induced autophagic vacuoles, double membrane autophagosome structures and the autophagy-related proteins P62, Bcl-2, ATG7 and LC3-II. In addition, the AKT-mTOR pathway was downregulated, while AMPK was upregulated in PS-PDT-treated cells. Inhibiting autophagy using chloroquine or by downregulating ATG7 using shRNA further upregulated apoptosis, suggesting autophagy was probably was protective to PS-PDT-treated tumor cells. *In vivo* relevance was demonstrated when a combination of chloroquine and PS-PDT significantly reduced the tumor size in a xenograft mice model. Our findings demonstrate that combination therapy using PS-PDT and autophagy inhibitors may be an effective approach to treating colorectal cancer patients.

## INTRODUCTION

Colorectal cancer (CRC) is the third most common cancer globally and the leading cause of cancer-related death. More than 1.2 million new cases and 600,000 colorectal cancer deaths are reported every year [1]. Surgical resection is the standard and primary treatment for early-stage CRC and it offers good long-term outcomes with a 5-year overall survival rate of about 65%. However, for patients with metastatic CRC, the overall 5-year survival rate drops dramatically to 5% [2]. Other therapeutic strategies employed for CRC treatment are radiation and chemotherapy. However, severe side effects are observed when these treatments are employed in advanced metastatic CRC patients [3–5]. Therefore, there is urgent need for novel modalities to clinically treat advanced metastatic CRC patients. Photodynamic therapy (PDT) is one such promising novel therapeutic strategy for CRC treatment. Patients, unsuitable for operation can be treated with endoscopic PDT due to the hollow structure of the colon. Moreover, PDT is a potent therapeutic approach for malignant and pre-malignant tumors and other diseases in the colon cavity [6, 7].

Typical PDT procedure includes the administration of a photosensitizing agent and activation of the photosensitizer by a light source of a specific wavelength in the presence of oxygen. Photochemical reactions triggered by this process result in generation of reactive oxygen species (ROS) selectively in the target tissues or cells [8]. The ROS are highly reactive that cause oxidative stress and DNA damage. These photochemical products are a result of high reactivity of ROS with the cellular components leading to destruction of cell organelles and other cellular components resulting in cell death either by apoptosis and/or necrosis [9]. PDT is highly suitable for CRC treatment and also very selective with the ability to eradicate tumors without destroying the adjoining connective tissues, thereby preserving the anatomical integrity of the colon in the patients [10].

Many different kinds of photosensitizers exist of which the second generation photosensitizer, Photosan-II (PS-II) is rather unique with the ability to accumulate more effectively and selectively in tumor cells. In addition it has better and stronger absorption at longer wavelengths (630 nm) and therefore considered as one of the most promising photosensitizers for future clinical PDT treatment [11, 12].

However, recent studies have suggested that signalling pathways such as autophagy are involved in resistance to PDT treatment [12]. Autophagy is a dynamic process that involves the transport of entire organelles and proteins in double-membrane vesicles termed autophagosomes [13]. Following fusion with lysosomes, the contents of the autophagosomes are digested and recycled for cellular energy and other anabolic processes. Autophagy is also crucial for eliminating damaged or aging intracellular components [14]. Autophagy is further shown to be involved in the pathophysiology of starvation, infection and tumorigenesis [15]. However the functional role of autophagy in cancers is controversial. While some studies have suggested that autophagy protects tumours, other reports have suggested that autophagy can induce cell death, especially in apoptosis-resistant cells [16].

Although previous studies have shown that autophagy can be induced by PDT and maybe linked to PDT resistance, its function in PDT has not been clearly elucidated [17, 18]. Researchers found that silencing of the autophagy gene *ATG7* increased photosensitization levels of mouse leukemia L1210 cells to photodynamic treatments [19]. However, in human breast cancer MCF-7 cells, silencing of *ATG7* enhanced resistance to PDT [20]. Collectively, these studies suggested that PDT could induce autophagy and apoptosis and that autophagy might play contradictory roles depending on the cell types and the type of photosensitizers [21]. Therefore, further studies are necessary to understand the role of autophagy in PDT treatment.

In this study, we tested the cytotoxic and anti-proliferative effects of PS-PDT on human colorectal SW620 and HCT116 cells. Then, we further investigated the signaling pathways that modulate both apoptosis and autophagy in SW620 and HCT116 cells in response to PS-PDT treatment. Finally, we studied the consequences of inhibiting autophagy during PS-PDT treatment using both *in vitro* and *in vivo* colorectal cancer models to gain therapeutic insights.

## RESULTS

### Cytotoxic and anti-proliferative effects of PS-PDT on human colorectal cancer cells

**Figure 1:**
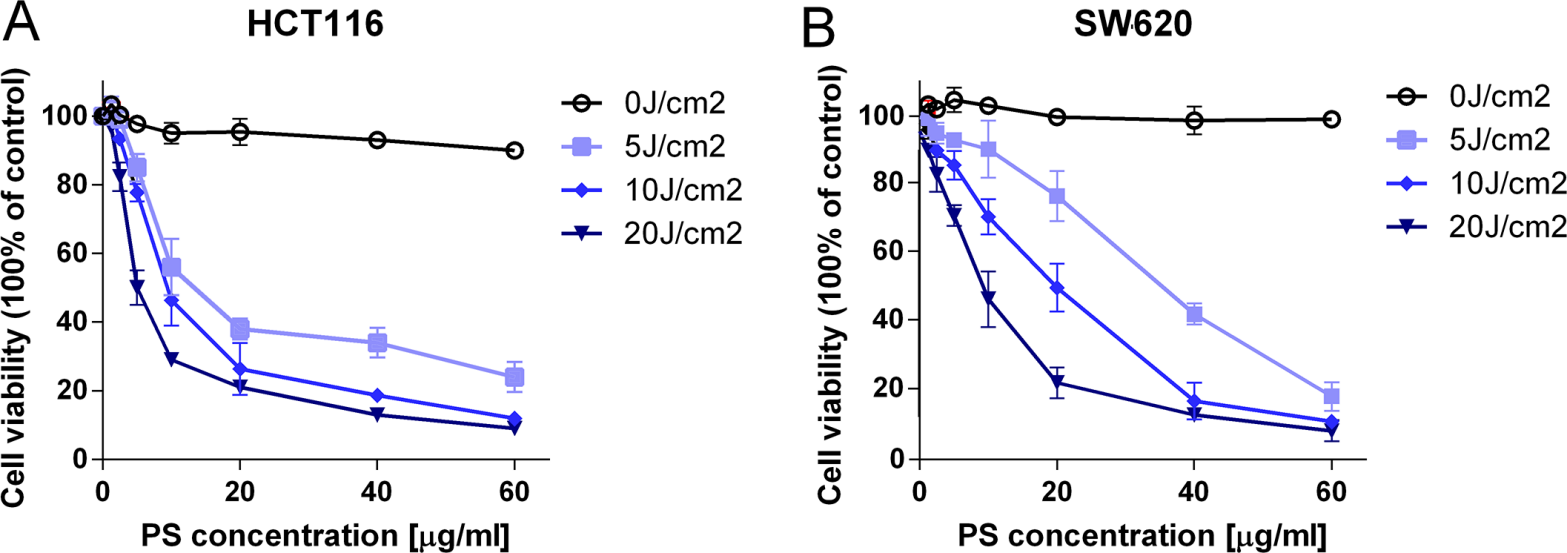
The cytotoxic and anti-proliferative effect of PS-PDT on human colorectal cancer cells The cell survival curves of HCT116 (**A**) and SW620 (**B**) in the presence of different doses of PS-II with or without irradiation. The data are expressed as mean ± sd from the results of three independent experiments. IC_50_, half maximal inhibitory concentration; J/cm^2^, joule per square centimeter for radiation exposure.

To evaluate the cytotoxic and antiproliferative effects of PS-PDT on human colorectal cancer cells, the HCT116 and SW620 cells were loaded with PS-II (1.25 to 60 μg/ml) for 4 h in darkness to allow for cellular absorption and intracellular accumulation, whereas, the blank cells were left untreated. Then, each of the treated cells were divided into two groups, of which, one was photodynamically irradiated (5, 10, 20 J/cm^2^) using a laser light of 630 nm wavelength, whereas, the other group was left untreated. The cells were then analyzed by the CCK-8 assay to determine the cytotoxic effects. No significant difference in viability were found between the blank and the control group (*p* > 0.05) for both the cell lines (PS-II < 40 μg/ml for HCT116; Figure 1A and 1B). At the highest PS-II dose of 60 μg/ml, we observed less than 10% inhibition in the control groups of both cell lines (Figure 1A and 1B) indicating that it was non-toxic. However, treatment with PS-II (> 5 μg/ml) followed by light irradiation (> 10 J/cm^2^) resulted in a sharp decline in viability of both HCT116 and SW620 cells compared to the control groups, with only around 58.4 ± 4.3% and 73.2 ± 4.9% viability for HCT116 and SW620 respectively after treated with 10 μg/ml PS-II and 10 J/cm^2^ irradiation (Figure 1A and 1B) (*p* < 0.01). This demonstrated the anti-proliferative and cytotoxic effects induced by light-activated PS-II in the tumor cells. The inhibition rate was dependent on the dose of PS-II used as well as the intensity of light (Table 1). We calculated the IC_50_ value from the cell survival curves constructed for each condition and found that IC_50_ decreased with an increase in both the PS-II concentration and the light intensity. Further, based on the comparison of the IC_50_ values, we found that HCT116 was more sensitive to PS-II than SW620 (Table 1).

**Table 1:** The IC_50_ value of PS-PDT in HCT116 and SW620 cells

	Cell lines		light intensity (J/cm^2^)		
		0	5	10	20
HCT116	IC_50_(μg/ml)	/	14.83 ± 1.65	11.08 ± 1.07	5.76 ± 0.62
	*R^2^*	/	0.9968	0.9876	0.9937
SW620	IC_50_(μg/ml)	/	27.58 ± 3.12	16.54 ± 2.09	8.32 ± 1.03
	*R^2^*	/	0.9863	0.9795	0.9948

The data expressed as mean ± sd from the results of three independent experiments; IC_50_ , half maximal inhibitory concentration; J/cm^2^, joule per square centimeter for radiant exposure; *R^2^*, The square of the correlation coefficient in the linear regression;

### PS-PDT triggered apoptotic cell death on human colorectal cancer cells

**Figure 2:**
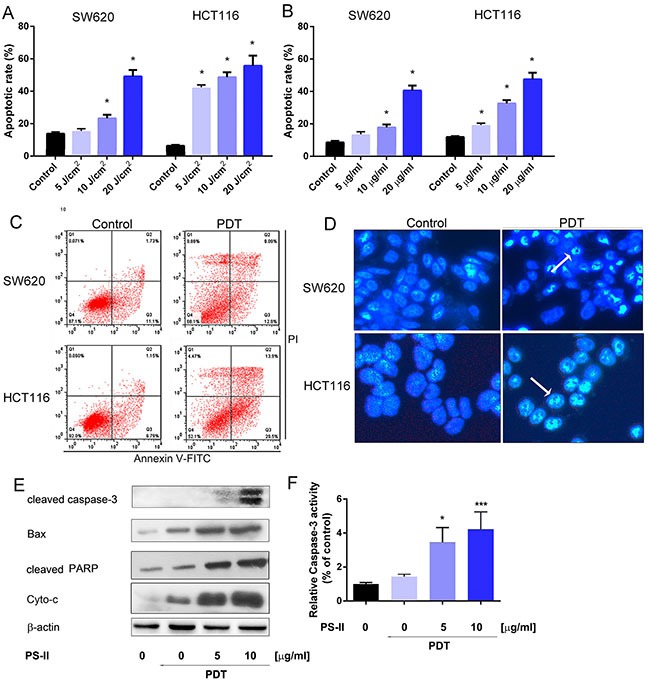
Apoptosis induction in HCT116 and SW620 cells by PS-PDT The apoptotic rate in HCT116 and SW620 cells was determined 24 h after treatment with various doses of light irradiation with a fixed PS-II (10 μg/ml) (**A**) or different concentrations of PS-II with a fixed intensity (5 J/cm^2^) (**B**). (**C**) FACS analysis of apoptosis in HCT116 and SW620 cells after treated with 10 μg/ml PS-II and 5 J/cm^2^ light irradiation. (**D**) Hoechst staining and analysis of the condensed nuclei by fluorescence microscopy (200 × magnification). Apoptotic cells are shown in blue and the peripherally clumped or fragmented chromatin is indicated by arrows. (**E**) Western blot analysis of cleaved Caspase-3, Bax, cleaved PARP and Cyto-c (cytochrome C). β-actin was used as a control. (**F**) The relative activity of caspase-3 was detected in different groups by using ELISA kit. Data are expressed as mean ± sd of three independent experiments. *p < 0.05, ***p < 0.01 vs. untreated control (HCT116 or SW620).

To investigate if the inhibition of growth in SW620 and HCT116 cells upon PS-PDT treatment was due to apoptosis, we quantitated apoptosis by flow cytometry using Annexin V-FITC/propidium iodide (PI) double staining. Our data showed that PS-PDT treatment significantly increased apoptosis in both SW620 and HCT116 cells at various PS-II doses and light intensities (*p* < 0.01; Figure 2A and 2B). The HCT116 cell line was more apoptotic compared to SW620 at different PS-II concentrations under fixed light intensity of 10J/cm^2^ (Figure 2A). The apoptotic rate was dose-dependent and increased with increasing PS-II concentrations in both the cell lines (Figure 2B). Also, the percentage of early apoptosis in the irradiated cells increased proportionately with enhanced light intensities. After the 10 μg/ml PS-II and 5J/cm^2^ irradiation treatments, the apoptotic rates for SW620 and HCT116 increased from 12.83% to 22.0% and 7.94% to 43.4%, respectively (Figure 2C and Table 2B). The classic morphological changes that occur during apoptosis like chromatin condensation, nuclear fragmentation and or degraded nuclei were monitored to further confirm apoptosis induced by PS-PDT treatment. Towards this, 24 h after PS-PDT treatment, cells were stained with Hoechst 33342 and visualized by microscopy (Figure 2D). The control cells showed relatively weak fluorescence with no visible nuclear changes, whereas the PS-PDT treated HCT116 and SW620 cells demonstrated typical apoptotic characteristics such as nuclear condensation and/or degraded nuclei (Figure 2D). In addition, western blotting analysis showed that PS-PDT treatment in SW620 cells upregulated the activated form of caspase-3, Bax, cleaved PARP and Cytochrome C (Figure 2E). Further, we observed a 3 fold increase in caspase-3 activity after PS-PDT treatment in dose dependent manner (Figure 2F). These results showed that PS-PDT induced apoptotic cell death in SW620 and HCT116 cells.

**Table 2:** The apoptotic rate induced by PS-II (10 μg/ml) and light irradiation (5J/cm^2^) in HCT116 and SW620 cells

Cell lines	Untreated controls			PS-PDT treated		
	Viable (Q4)	Total apoptosis (Q2 + Q3)	Necrosis (Q1)	Viable (Q4)	Total apoptosis (Q2 + Q3)	Necrosis (Q1)
HCT116	87.1% ± 4.56	7.94% ± 0.56	0.060% ± 0.01	52.1% ± 2.61	43.4% ± 2.08	4.47% ± 0.12
SW620	92.0% ± 4.91	12.83 ± 0.94	0.071% ± 0.01	68.1% ± 3.01	22.0% ± 1.87	9.85% ± 0.38

The data are expressed as mean ± sd from the results of three independent experiments; Q, quadrant.

### PS-PDT induced autophagy

**Figure 3:**
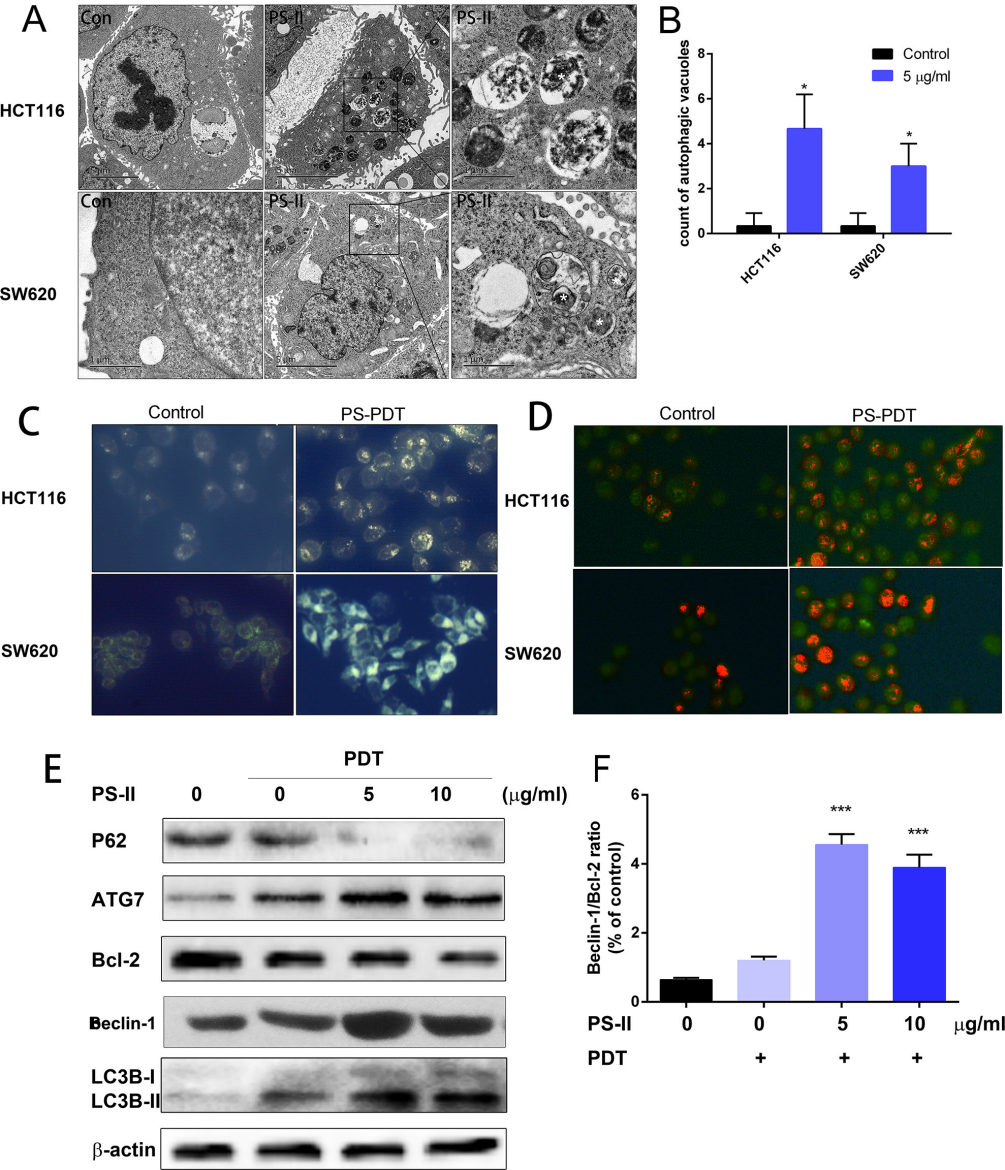
PS-PDT induced autophagy in SW620 and HCT116 cell lines The SW620 and HCT116 cells were treated with 5 μg/ml PS-II followed by a 10 J/cm^2^ illumination. (**A**) TEM analysis of the autophagic vacuoles. Representative autophagic vacuoles in SW620 and HCT116 were detected by transmission electron microscopy. Typical specific double-membrane structures packaged by part of the cytoplasm and the organelles are indicated by the asterisk. (**B**) Quantitative analysis of the number of autophagic vacuoles for different treatment groups. (**C**) Visualization of the autophagy activation in SW620 and HCT116 by monodansylcadaverine (MDC) staining. (**D**) Acridine orange staining showing the accumulation of acidic vesicular organelles as bright red fluorescence in PS-PDT treated HCT116 and SW620 cells. (**E**) Western blotting analysis of the autophagy related protein p62, ATG7, Bcl-2, LC3B-I and II in cell samples after PDT treatment. (**F**) The ratio of Beclin-1 and Bcl-2 expression was calculated according to the western blotting results. *p < 0.05, ***p < 0.01 compared with untreated control group. The data are expressed as mean ± sd from the results of three independent experiments.

Subsequently, we investigated if PS-PDT treatment initiated autophagy in the SW620 and HCT116 cells. First, we examined the SW620 and HCT116 cells that were treated with PS-PDT treatment for autophagic vacuoles using TEM, which is the classical approach to determine autophagy [22]. Double membrane-bound vacuoles that are typical of autophagosomes were observed in the cytoplasm and organelles of the SW620 (Figure 3A, lower panel) and HCT116 (Figure 3A, upper panel) cells within 1 h after PS-PDT treatment. The control SW620 (Figure 3A, lower panel) and HCT116 cells (Figure 3A, upper panel) demonstrated normal morphology of both the cytoplasm and the organelles and autophagic vacuoles were not observed. The autophagic vacuoles that were found in the PS-PDT treated SW620 and HCT116 cells had engulfed bulk cytoplasm and cytoplasmic organelles. Also, we found a quantitative increase in the autophagic vacuoles following PS-PDT treatment (Figure 3B).

To further characterize the autophagic response upon PS-PDT treatment, we assessed the treated cells by monodansylcadaverine (MDC) and acridine orange staining. We observed accumulation of the auto-fluorescent dye, MDC, in both the PS-PDT treated SW620 and HCT116 cells compared to the untreated cells showing that PS-PDT induced the formation of autophagic vacuoles (Figure 3C). Further, the cells were stained with acridine orange to quantitate the formation of acidic vesicular organelles (AVOs) and the subsequent fluorescence microscopy analysis showed that PS-PDT treated SW620 and HCT116 cells had significantly bright red fluorescence indicating an accumulation of AVOs compared to controls (Figure 3D). Then, we checked the effect of PS-PDT on the autophagy signaling pathway in the treated SW620 and HCT116 cells by western blotting to quantify critical autophagy related proteins such as p62, Beclin-1 and LC3. As shown in Figure 3E, there was a dose-dependent decrease p62 and Bcl-2 in PS-PDT treated cells compared to the controls at 24 h. Since p62 is involved in the autophagic sequestration process, its down-regulation significantly affects the autophagy process [23]. Further, we also traced the conversion of LC3-I to the active form of LC3-II, a marker for autophagic vesicles and autophagic activity. When autophagy-related proteins conjugated to phosphatidylethanolamine (PE) and attached to LC3-I to form LC3-II and is correlated with autophagic reflux [24]. As shown in Figure 3E, there was a dose-dependent increase in the LC3-II form in the PS-PDT treated SW620 cells at 24 h. The increase in the Beclin-1 to Bcl-2 ratio after PS-PDT treatment indicated a promotion of autophagy and a suppression of apoptosis (Figure 3F). These results further confirmed that PS-PDT induced the autophagy in HCT116 and SW620 cells.

### AMPK activation and AKT down-regulation promoted autophagy in SW620 cells

**Figure 4:**
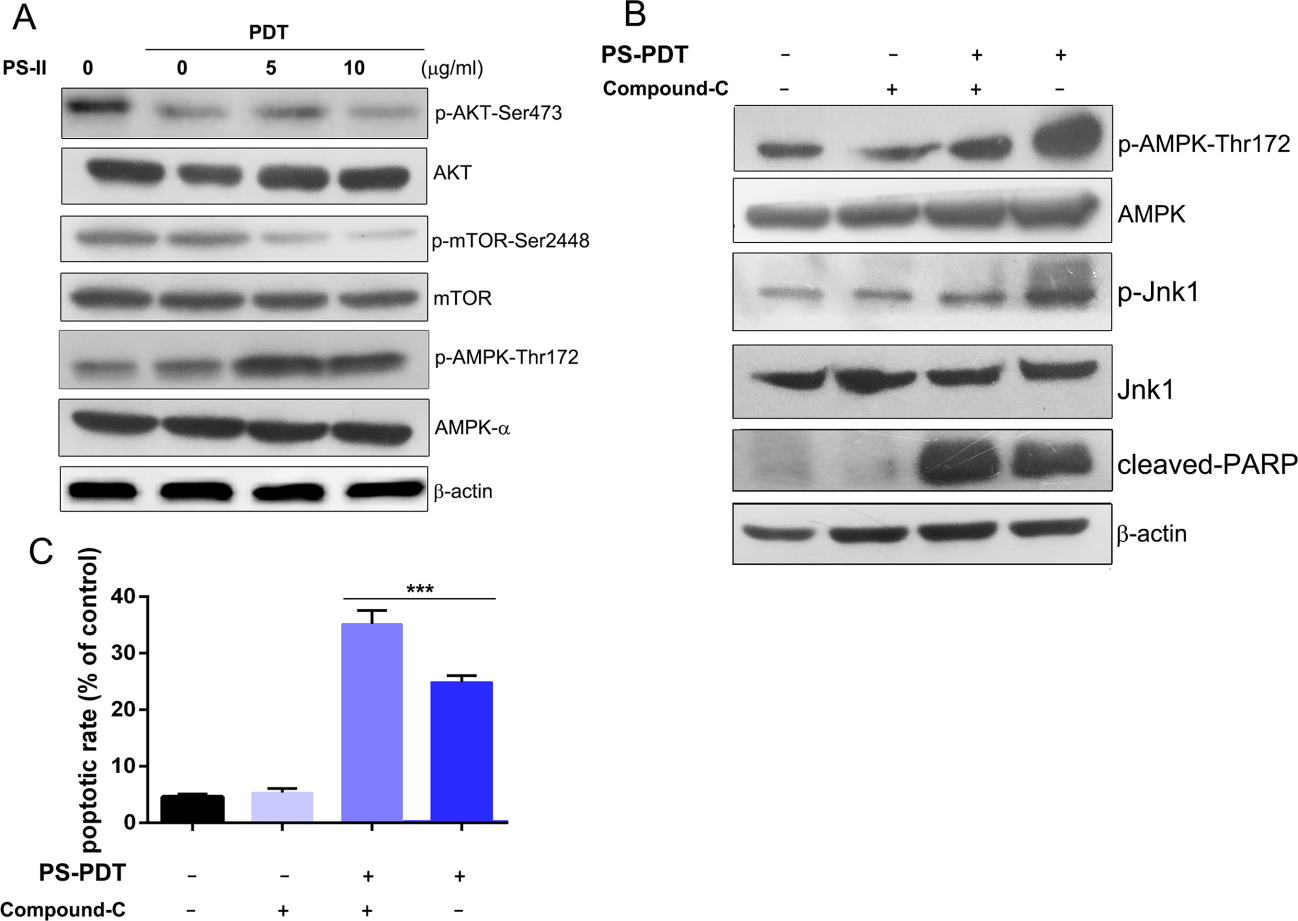
Analysis of AMPK activation and AKT/mTOR pathway inhibition in PS-PDT-treated SW620 cells The SW620 cells were treated with 5 mg/L and 10 mg/L PS with illumination of 5J/cm^2^ and were incubated for 24 h. (**A**) Western blot analysis of the phosphorylation of AKT-Ser473, AMPK-Thr172 and mTOR-Ser2448. The total protein of AKT, AMPK and mTOR are also shown. (**B**) Western blot analysis of the phosphorylation of AMPK (Thr172), Jnk1 (Thr183/Tyr185), cleaved PARP was shown after the AMPK specific inhibitor compound C treatment the phosphorylation of AMPK that was induced by PS-PDT. (C) Apoptosis in SW620 cells after PS-PDT treatment in combination with compound C was analyzed using Annexin V-FITC/PI staining. ***p < 0.01 compared with untreated control group. The data are expressed as mean ± sd from the results of three independent experiments.

The AKT-mTOR (mammalian target of rapamycin) pathway is vital in regulating the balance between apoptosis and autophagy [25]. Therefore, we investigated the impact of PS-PDT treatment on both mTOR and AKT. By western blot analysis, we found that active mTOR declined at 24 h after PS-PDT treatment (Figure 4A). In addition, phosphorylated AKT1 (-Ser473) was downregulated in PS-PDT treated SW620 cells suggesting suppressed AKT kinase activity and the activation of mTOR (Figure 4A). Our results also suggested that PS-PDT treatment may modulate the downstream targets of AKT.

AMP-activated protein kinase (AMPK) is a key energy sensor that regulates cellular metabolism and autophagy in cancer cells and normal cells [26]. We found increased AMPK phosphorylation at Thr172 in a dose dependent manner (Figure 4A). We postulated that AMPK may be involved in the PS-PDT-induced autophagy pathway. To determine this, we used the AMPK specific inhibitor, compound-C, to block the activation of AMPK and its downstream substrate Jnk1 with an increase of cleaved PARP. We also observed significantly higher apoptosis in SW620 cells treated with a combination of PS-PDT and compound C compared to the controls (*p* < 0.05; Figure 4B). We also observed significantly higher apopotic cells in SW620 treated with a combination of PS-PDT and compound C compared to the controls (*p* < 0.05; Figure 4C). Taken together, our data shows that the activation of AMPK and down-regulation of AKT pathway regulate autophagy upon PS-PDT treatment.

### Autophagy inhibition enhanced the apoptosis induced by PS-PDT

**Figure 5:**
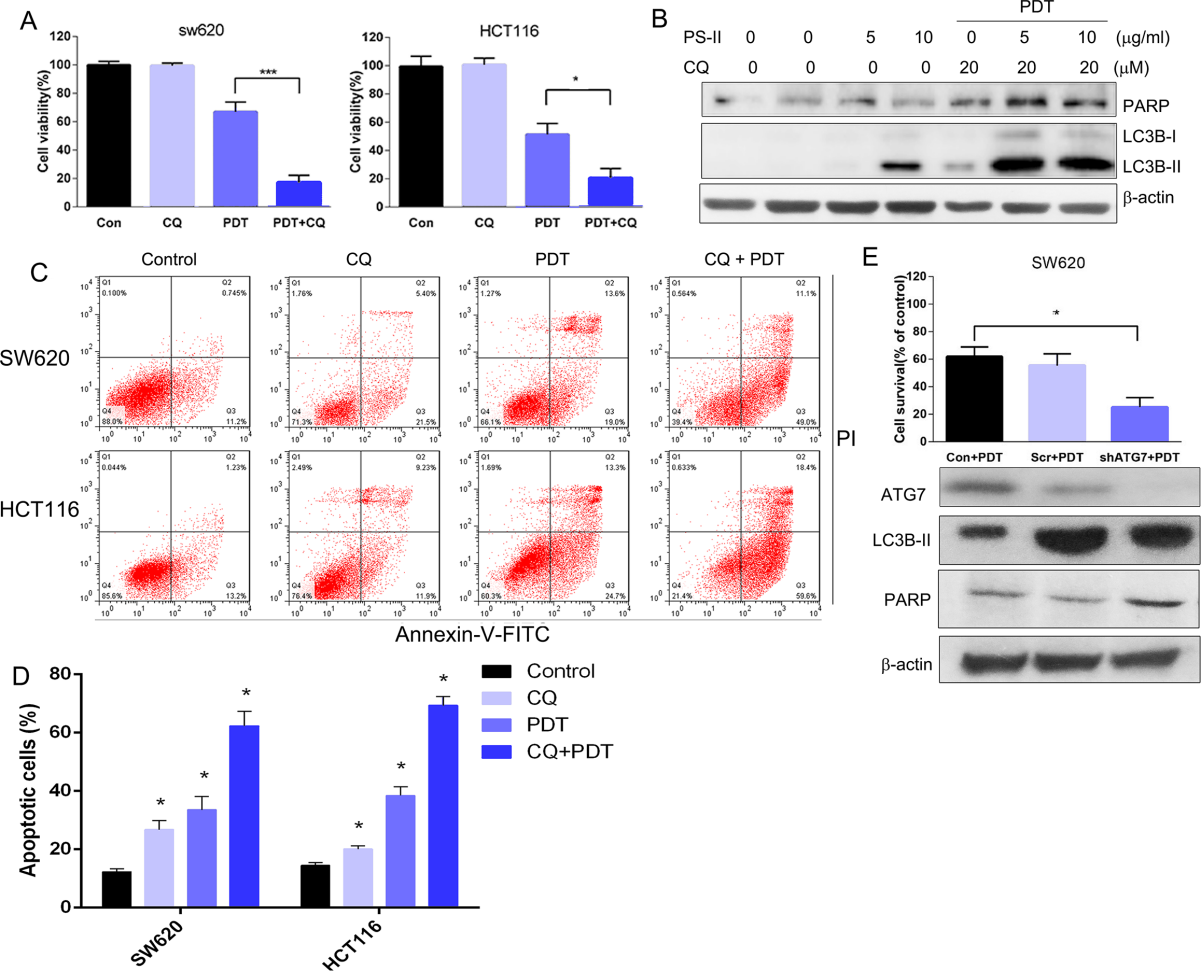
Autophagy inhibition enhanced the PS-PDT-induced apoptosis in SW620 and HCT116 cells (**A**) The cell viability of SW620 and HCT116 cells with or without CQ was determined at 24 h post-PDT by the CCK-8 assay. (**B**) Western blot analysis of Caspase LC3B-I, LC3B-II and cleaved PARP expression after PS-PDT treatment in SW620 cells. 20 μM CQ was used to treat the cells to block the autophagy induced by PDT. (**C**) FACS analysis of apoptosis based on AnnexinV/PI staining in different treatment groups. (**D**) Apoptotic rate from each group. (**E**) The down-regulation of autophagic related protein ATG7 expression by specific shRNA in SW620 cells. The cell viability of control, ATG7^–^/^–^ and scramble shRNA transfected SW620 cells were determined using the CCK-8 assay. The expression of ATG7, LC3B-II, PARP and β-actin proteins were verified western blot assay. *p < 0.05, ***p < 0.01 compared with untreated control group. The data are expressed as mean ± sd from the results of three independent experiments.

To evaluate the function of autophagy induced by PS-PDT treatment, we used chloroquine (CQ), a specific autophagy inhibitor, or knocked down ATG7 that is involved in autophagy using a specific shRNA in order to investigate the consequences of downregulating autophagy on PS-PDT treatment outcomes in SW620 and HCT116 cells. We observed that combination of CQ increased cell cytoxicity of the PS-PDT in treated cells in comparison to either CQ or PS-PDT treated cells (Figure 5A). Further, by western blotting, we found that CQ enhanced the PS-PDT induced LC3-II expression levels compared to PS-PDT treatment alone (Figure 5B). Similarly, the levels of cleaved PARP were further increased in PS-PDT and CQ co-treated cells compared to the PS-PDT treated cells indicating that inhibition of autophagy enhanced PS-PDT-induced apoptosis (Figure 5B). The cell viability of the PS-PDT and CQ pre-treatment group was significantly lower compared to PS-PDT without CQ pre-treatment (*p* < 0.05). Since these data suggested enhanced apoptosis, we quantified apoptosis by Annexin V-FITC/PI staining and found enhanced apoptosis in the PS-PDT and CQ co-treatment group (*p* < 0.05) (Figure 5C and 5D). We then tested if down-regulation of an essential autophagic gene, *ATG7*, in SW620 cells would show similar results as chloroquine treatment. We transfected ATG7 shRNA (shATG7) along with scramble shRNA (scr) into SW620 cells and detected the protein expression changes in these cells. As expected, we observed that ATG7 downregulation resulted in reduced autophagy in the PS-PDT-treated cells (Figure 5E) Also, *ATG7* knockdown resulted in a 40% decrease in cell viability in PS-PDT treated cells compared to the appropriate controls (*p* < 0.05; Figure 5E). Taken together, these results suggest that autophagy may play a pro-survival role in the PS-PDT treatment in colorectal cancer cells.

### *In vivo* therapeutic efficacy of combined PS-PDT and chloroquine treatment

**Figure 6:**
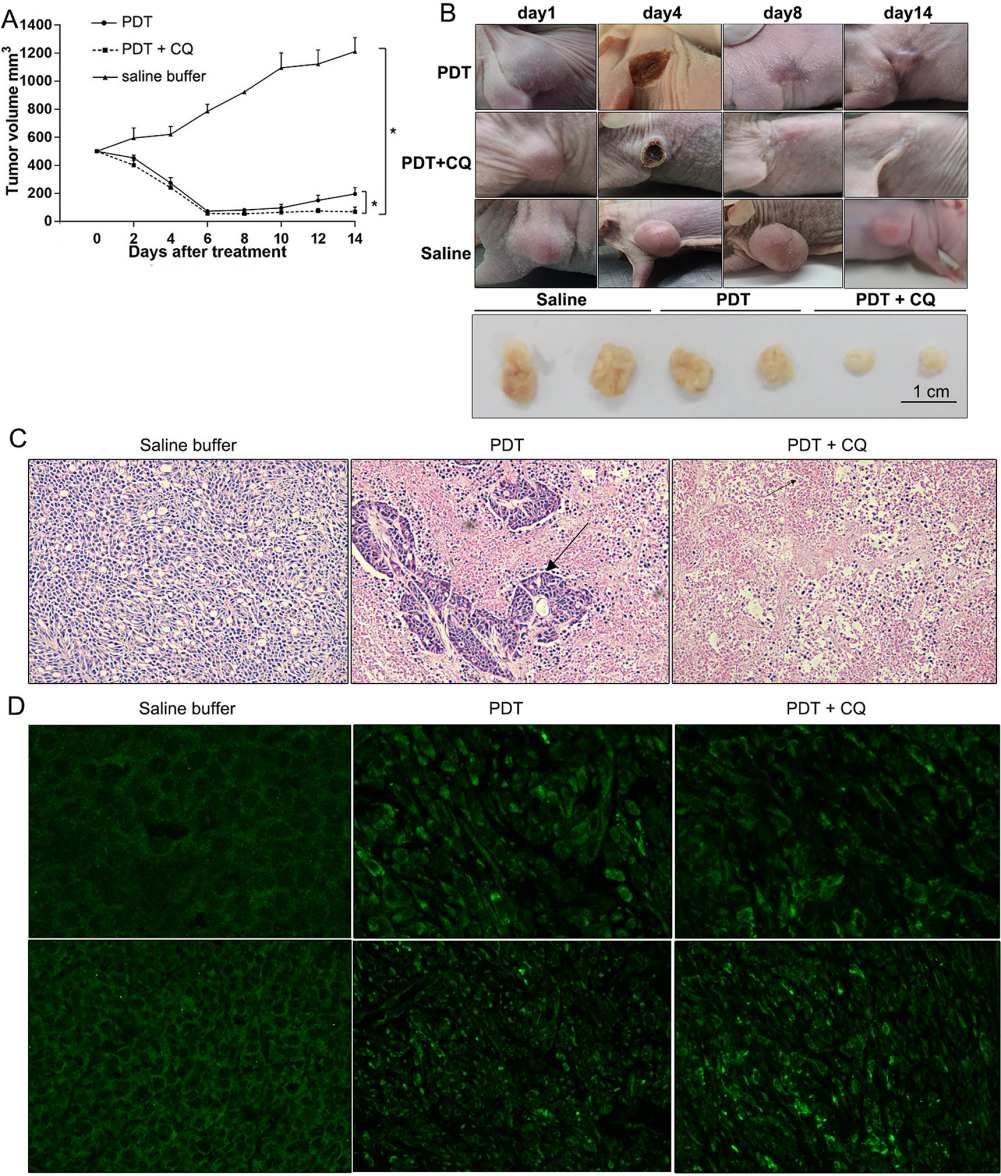
In vivo therapeutic efficacy of PS-PDT with chloroquine (CQ) CRC xenografts derived from SW620 cells were treated with control (saline buffer), PS-PDT alone (PS-II 10 mg/kg) or combination therapy (PS-PDT with CQ) for 13 days, CQ (60 mg/kg/twice daily) (**A**) Tumor growth curve of the SCID mice bearing SW620 cells. (**B**) Representative of each group during the treatment. (**C**) Hematoxylin and eosin (H&E) staining of subcutaneous implanted SW620 cells from each group. The arrows indicate the typical apoptotic cells that are shrunken with condensed cytoplasm. Magnification, 40×. (**D**) Immunostaining of endogenous LC3 in the tumor tissues of each group. Magnification, 40× (upper panel); 10× (lower panel). The results represent the mean ± sd (*n* = 6). *p < 0.05.

Finally, we investigated the *in vivo* therapeutic efficacy of PS-PDT treatment in combination with autophagy inhibitors by xenografting SW620 cells in SCID mice. The tumor-bearing mice were divided into three different groups and were administrated either saline buffer (control group), PS-PDT alone or PS-PDT with PDT CQ every 2 days for six consecutive cycles. The tumor size and volume from each group of mice was measured and recorded every 2 days until the 14th day (Figure 6B). The results indicated that the tumor volume was significantly reduced in the PS-PDT and CQ combination group (Figure 6A–6B), suggesting an *in vivo* therapeutic efficacy of PS-PDT. These results implied that combination treatment of autophagy inhibitor and PS-PDT may reduce the rate of recurrence for CRC.

We then analyzed the pathophysiological changes of the transplanted tumors in host mice by histological examination and immunohistochemistry (IFC) assay (Figure 6C). The tumour sections from mice 48 h after treatment were fixed and stained with H&E (hematoxylin and eosin) and examined under light microscope. A significantly increased cell apoptosis and necrosis was observed in the tumours receiving a combination of CQ and PS-PDT with the typical shrunken apoptotic cells and condensed cytoplasm observed in the PS-PDT and PS-PDT plus CQ groups. This result indicated that CQ promoted the PS-PDT-induced apoptosis *in vivo*.

To further confirm whether CQ blocked PS-PDT-induced autophagy, we detected the distribution of LC3, an autophagy marker by immunohistochemistry. We observed that whereas the fluorescence of LC3 showed a diffuse nuclear and cytosolic distribution in control group (saline buffer treated), it demonstrated a punctate distribution in PS-PDT treated cells reminiscent of induced autophagy (Figure 6D). The CQ treatment was found to inhibit the formation of the punctuate LC3.

Our results implied PS-PDT induced substantial autophagosomes within the tumor tissue *in vivo* that were decreased by CQ treatment in combination with PS-PDT. Therefore, we postulate that when the mice were treated with PS-PDT, the autophagy induced within the cancer cells resisted the chemotherapeutic drug-induced apoptosis and therefore autophagy inhibition further enhanced cell death and hence reduced the tumor sizes. Therefore, our study postulates that combining PS-PDT treatment with autophagy inhibitors such as CQ would improve the clinical efficacy of PDT in colorectal cancer treatment.

## DISCUSSION

Photodynamic therapy is a clinical approved therapeutic procedure that has been tested for treatment of multiple cancers in recent decades [27]. It can inhibit cancer cell proliferation by causing oxidative stress and DNA damage through reactive oxygen species (ROS). Excessive oxidative stress and DNA damage can induce several cellular pathways like apoptosis and autophagy, which affect the therapeutic efficacy of photosensitizers in many cancer cell lines [28]. Apoptosis has been reported in PDT-mediated tumor inhibition for several cancer cell lines [29–31]. Autophagy is a homeostatic cellular recycling mechanism that has been designated as programmed cell death type II [32]. Although there are a few studies that have reported PDT-induced autophagy, the mechanism still needs to be understood in detail [33–36]. A recent study suggested that autophagy promoted resistance to photodynamic therapy-induced apoptosis selectively in colorectal cancer stem-like cells [10]. Therefore, the interplay between autophagy and apoptosis during PDT treatment could possibly affect the efficacy of PS-PDT.

Therefore, to get a better understanding of the role and interplay between autophagy and apoptosis during PDT treatment, we investigated the anti-cancer effects of PS-PDT on human colorectal cells. Our data suggests that PS-PDT significantly inhibited the cell proliferation of both, the human colorectal cancer cells, in a dose-dependent manner. Subsequently, we confirmed that PS-PDT significantly induced apoptotic cell death based on the increased ratio of the Bax/Bcl-2 proteins, the activation of caspase-3 and PARP cleavage in PS-PDT-treated cells. Meanwhile, we also found that PS-PDT treatment dramatically increased expression of typical autophagy-specific proteins such as LC3-II, ATG7 in human colorectal cells. Further, our studies demonstrated the accumulation of autophagic vacuoles by MDC staining and double-membrane autophagic vacuoles by TEM. We also confirmed the development of the acidic vesicular organelles by acridine orange staining suggesting formation of the autophagolysosomes. These data indicated that both apoptosis and autophagy were triggered by PS-PDT in SW620 and HCT116 cells. Thus, we postulated that understanding the regulation of the crosstalk between apoptosis and autophagy would help uncover the cytotoxic and anti-proliferative effects of PS-PDT.

To further characterize the regulation of autophagy and apoptosis in PS-PDT treatment, we examined the status of the signaling pathways after the PS-PDT treatment. AKT is a key regulator of both apoptosis and autophagy through phosphorylation of proteins including Bad, Bax and mTORC1 substrates and may be critical in PS-PDT-induced apoptosis and autophagy as reported by previous studies [37]. Our results showed that inhibition of Akt phosphorylation and downstream mTOR signaling after PS-PDT. We found that down-regulation of Akt phosphorylation and mTOR was negatively correlated with the accumulation of cleaved LC3-II after PS-PDT treatment. This was probably caused by the negative feedback activation of mTOR onto Akt [38]. We also observed up-regulation of key AKT substrates that regulate apoptosis, namely, Bcl-2 and Bax and down-regulation of autophagy regulator, p62. Therefore, these data clearly indicated that the AKT-mTOR signaling pathway plays a critical role in the PS-PDT-induced autophagy and apoptosis We also noticed that AMPK might be involved in PS-PDT induced autophagy.

The role of autophagy is still controversial and has been documented to either promote or prevent cell death under various physiopathological conditions [39, 40]. On one hand, autophagy can prevent intracellular DNA damage by eliminating the damaged proteins and organelles in the cells that modulate the genetic instability, a driving force of tumorigenesis [41]. On the other hand, autophagy that is activated by various kinds of stimuli has been shown to promote tumor cell survival under stress [42]. Also, autophagy has been shown to induce cell death (autophagic programmed cell death), if cells recycled damaged constituents that affected cellular homeostasis [43, 44]. Therefore, to determine the role of autophagy induced by PS-PDT, we used CQ, a late-stage autophagy blocker to disruption of autophagy and investigated the cell death signalling and cross-talk between the different cell death pathways. As expected, CQ significantly enhanced the PS-PDT-induced reduction of cell viability compared to cells undergoing only PS-PDT treatment. This demonstrated that autophagy induced by PS-PDT lead to colorectal cancer cell survival instead of cell death.

Many therapeutic treatments have also validated that modulating autophagy signaling could affect their efficacy. Several previous studies reported that PDT combination with CQ or other autophagy inhibitory drugs could promote the apoptotic cell deaths [45]. However, such results have not been tested or validated on PS-PDT treatment. Thus, we used the CQ or *ATG7* shRNA to block the autophagy to test if PS-PDT treatment promoted apoptotic cell death. As expected, we observed increase of cleaved PARP, a typical marker of cell apoptosis and increased apoptotic cell death by FACS analysis in PS-PDT and CQ co-treated cells. More importantly, we found similar results in the xenograft tumor model with a significant reduction in tumour volumes after PS-PDT and CQ combination treatment. Taken together, these results suggested that autophagy modulation may be helpful in promoting the effects of PS-PDT. Previous studies have reported that inhibition of autophagy either by an autophagy inhibitor like CQ or silencing of critical genes such as *ATG7* can result in apoptotic cell death due to energy deprivation in cells [46, 47]. In our results, CQ enhanced the anti-tumor effect of PS-PDT in colorectal cancer cells in a very similar manner, although further detailed studies will be needed to understand the mechanism in greater detail.

In conclusion, we demonstrate that both autophagy and apoptosis are triggered in response to PS-PDT treatment in colorectal cancer cells. We also demonstrate that the AKT/mTOR signalling pathway is modulated in response to PS-PDT and that in turn regulates downstream apoptosis and autophagy targets that affect the balance of the two key cellular mechanisms. We further showed that autophagy inhibition during PS-PDT treatment enhances apoptotic cell death in CRC cells both *in vitro* and *in vivo*. Taken together, our study provides experimental evidence that modulation of autophagy may be necessary to improve the efficacy of PS-PDT in human colorectal cancer therapy.

## MATERIALS AND METHODS

### Cell culture and maintenance

The human colorectal cancer cell lines, SW620 and HCT116 that were purchased from the Cell Center of the Xiangya School of Medicine (Hunan, China) were grown in RPMI 1640 medium (GE Healthcare Life Sciences, USA) supplemented with 10% FBS (fetal bovine serum) and 1% penicillin-streptomycin mixture solution (Thermo Fisher Scientific, USA) at 37°C in a humidified incubator maintained with 5% CO_2_.

### Preparation of photosensitizers

The photosensitizer, polyhematoporphyrin (Photosan-II, PS) was purchased from Seehof Laboratorium F&E GmbH (Wesselburenerkoog, Germany). A stock 1 mg/mL PS-II solution was prepared in phosphate-buffered saline (PBS) and maintained in the dark at 4°C for less than one week and diluted in growth medium prior to use.

### Photosensitization and cell treatments

The cells that were about 80% confluent were pre-treated with different concentrations of PS-II in culture medium for 4 hours after they reached approximately 80% confluence. The cells were then exposed to various light intensities for 60 seconds under a 630 nm laser emitted by a semiconductor laser device (Laser Medical Tech Co., Ltd., Shenzhen, China). Then, the cells were transferred into fresh medium (with 10% FBS) and incubated in 37°C for 24 h for further experiments. For autophagy related experiments, the autophagy blocker chloroquine (Sigma-Aldrich, Germany) was added to culture medium before sensitization and then the protocol followed as described above.

### Cell proliferation assay

The cell viability was measured using the CCK-8 assay kit (Dojindo Laboratories, Japan). HCT116 and SW620 cells (2 × 10^4^) were first seeded into each well of a 96-well plate and grown overnight for PS-PDT treatment. After treatment, the culture medium was removed and replaced with 100 μl of fresh medium containing 10 μl of CCK-8 solution in each well and the cells were incubated at 37°C for 2 h. The plates were read in an Infinite 200 pro multi-mode microplate reader (Tecan Switzerland) at 450 nm and the viable cell numbers in each well were measured in comparison to appropriate controls. Caspase-3 (active) ELISA Kit, Human was purchased from thermofisher scientific (KHO1091).

### Flow cytometry assay

Cell apoptosis was quantified for each condition using the AnnexinV-FITC/PI double staining kit (Roche, Switzerland). Briefly, the HCT116 and SW620 cells (1 × 10^5^ per/well) were cultured overnight in 12-well plates. After the treatment, the cells were trypsinized, collected by centrifugation and washed twice in sterilized PBS. The cells were then resuspended in 500 μl binding buffer and stained with 5 μl annexin V-FITC plus 5 μl of PI and incubated in the dark for 10 minutes at room temperature followed by analysis in a BD FACScan™ flow cytometer. The AnnexinV-FITC^+^/PI^-^ and AnnexinV-FITC^+^/PI^+^ cell populations were considered as the apoptotic cells.

### Hoechst 33342 staining

Cells (1 × 10^5^ cells/ml) were seeded in 6-well plates and incubated for 24 h. After PS-PDT treatment, the cells were stained with 5 μg/ml Hoechst 33342 (Sigma Aldrich, Germany) for 5 mins at 37°C. The cells were then washed twice in PBS to remove excess dye and observed under a fluorescence microscope (Eclipse E600 from Nikon, Japan) with 330–380/498 nm excitation/emission wavelengths, respectively.

### Monodansylcadaverine (MDC) staining

The autophagic vacuoles were quantified in cells after the PS-PDT treatment using the monodansylcadaverine (MDC) stain (Sigma Aldrich, Germany). Two hours post-irradiation, the cells were stained with 0.05 mM MDC in PBS for 15 min at 37°C, washed with PBS, followed by fluorescence microscopy (excitation/emission wavelengths: 335/498 nm).

### Acridine orange staining

The formation of acidic vesicular organelles (AVOs) in the cells was detected by staining the cells after PS-PDT treatment with the fluorescent dye, acridine orange (Sigma, Germany). Briefly, 2 h after the PS-PDT treatment, the cells were stained with 1 mM acridine orange in PBS containing 10% FBS at 37°C for 15 minutes and observed under fluorescence microscopy using the excitation wavelength at 488 nm and the emission wavelengths at 510–536 nm (green) and 650 nm (red).

### Western blotting

For western blot analysis, the SW620 cell samples after the PS-PDT treatment were lysed in 1× RIPA (radio-Immunoprecipitation assay) lysis buffer (Roche, Switzerland) containing a cocktail of the protease and phosphatase inhibitors for 30 mins. Cell lysates were clarified through centrifugation at 13,000 × g for 30 mins at 4°C. The supernatant was collected and the total protein concentration for each sample was determined by a BCA (bicinchoninic acid) assay kit (Beyotime Biotechnology,Shanghai, China). 25 μg of total protein from all samples were loaded onto a 10–15% sodium dodecyl sulfate polyacrylamide gel (SDS-PAGE) for electrophoretic separation. Then, the proteins on the gel were transferred onto a PVDF (polyvinylidene difluoride) membrane, blocked in 5% non-fat milk for 2 h at room temperature and incubated overnight with primary antibodies at 4°C. The membrane was then washed with TBS-T buffer (Tris-buffered saline, 0.1% Tween-20) and incubated with the secondary antibody for 2 h at room temperature. The immunoblots on the membranes were visualized and enhanced by the Pierce ECL western blotting substrate kit (Thermo Fisher Scientific, USA). The primary antibody cleaved caspase-3 (#9661), p62 (#5114), Bax (#2772), Bcl-2 (#4223), ATG7 (#2631), LC3 (#4108), PARP (#9542), phos-AKT-Ser473 (#9271), AKT (#9272), SAPK/JNK1 (Thr183/Tyr185) (#9251), JNK1 (#9252), Beclin-1 (# 3738), cytochrome C (#4272), phos-mTOR-Ser2448 (#2971), mTOR (#2972) and β-actin (#4976) were all purchased from Cell Signaling Technology (CST, Danvers, MA, USA). The goat anti-rabbit and goat anti-mouse secondary antibodies were purchased from Abcam (Shanghai, China). AMPK inhibitor Compound C (CAS: 866405-64-3) (Sigma-Aldrich, Germany) was resolved in DMSO.

### Transmission electron microscopy (TEM) analysis

For transmission electron microscopy (TEM) analysis, the HCT116 and SW620 cells were seeded into 6-well plates at a density of 5 × 10^5^ cells per well and fixed in 2.5% glutaraldehyde for 6 h after PDT treatment. The fixed cells were post-fixed with a 1% OsO4 buffer, dehydrated by passing through the graded alcohol steps and flat embedded in EPON^TM^ resin. The cell samples were cut into ultra thin sections (100 nm) and stained with 3% lead citrate plus uranyl acetate and observed under a CM20 electron microscope (Phillips, Holland).

### Cell shRNA interference

SW620 cells (1 × 10^5^ cells/well) were seeded in 24-well plate for 24 h prior to transfection. shRNA ATG7 sequences for the sense strand (5'-GCCTGCTGAGGAGCTCTCCAT-3') and the antisense strand (5'-AAGGAAGAGCTGTGACTCC-3'). The control and ATG7 shRNAs (Sangon Biotech, Shangai, China) were diluted in 5 μL Opti-MEM medium with reduced serum. The transfections were performed according to the instructions in the Cells transfection kit Lipofectamine^®^ 2000 (Invitrogen, USA). Further experiments were carried out 48 h after transfection.

### *In vivo* tumor inhibition in Xenograft mouse SW620 cells

All animal experiments were performed as approved by the Institutional Animal Care and Use Committee of the Central South University. Four week old female BALB/c nude mice were purchased from the Institute of Laboratory Animal Sciences, Chinese Academy of Medical Science (Beijing, China). The SW620 cells (1 × 10^7^ cells) in 100 μL PBS were subcutaneously injected into the mice at the axilla. Tumor sizes were measured with calipers and the tumor volume was calculated using the formula, V = a × b × c ×π × 4/3 (a: long diameter of the tumor; b: short diameter of the tumor; c: tumor height) [48]. The mice with tumors (approx. 50 mm^3^ ) were randomly divided into three groups (*n* = 6), (1) A negative control group that was treated with saline buffer; (2) A photosensitizer treated group and (3) A group treated with a combination of the photosensitizer with 50 mg/kg chloroquine. The experiments were performed at days 0, 2, 4, 6, 8, 10 and 12 for each group. Photosan-II solution was injected *via* peritoneal cavity at a single dose of 10 mg /kg. Four hours after treatment, mice were irradiated with a 630 nm semiconductor laser (630 nm, 100 mW/cm^2^ × 600 s). Tumor volumes were measured every 3 days after PDT treatment and the mice were sacrificed 14 days after treatment.

### Histopathology evaluation

The human colorectal cancer samples derived from the xenograft mouse models at the final stage of the experiments were fixed in 10% neutral buffered formalin (NBF) containing 4% formaldehyde, embedded in paraffin and sectioned at a thickness of 4 μm. Further, after deparaffinization, the sections were stained with hematoxylin-eosin (H&E) or used directly for immunofluorescence histochemistry assays.

### Statistical analysis

The data were expressed as the mean ± standard deviation (SD) from the results of at least three independent experiments. Student’s-*t* test and one-way analysis of variance (ANOVA) was used to evaluate statistical significance between the groups with a *p-value* less than 0.05 or 0.01 considered as significant (*) or very significant (***), respectively. All the statistical analysis was performed using the SPSS (Statistical Product and Service Solutions) software version 18.0.
